# A qualitative review of misinformation and conspiracy theories in skin cancer

**DOI:** 10.1111/ced.15249

**Published:** 2022-06-15

**Authors:** Cathal O'Connor, Siobhán Rafferty, Michelle Murphy

**Affiliations:** ^1^ Department of Dermatology South Infirmary Victoria University Hospital Cork Ireland; ^2^ Department of Medicine University College Cork Cork Ireland

## Abstract

Misinformation on diseases and treatments is a worldwide threat and can lead to worse outcomes for patients with skin cancer. The aim of this study was to qualitatively assess the content of online misinformation related to skin cancer. Searches were performed via PubMed and Google using the terms ‘skin cancer’ OR ‘melanoma’ OR ‘non‐melanoma skin cancer’ OR ‘SCC’ OR ‘BCC’ AND ‘misinformation’ OR ‘disinformation’ OR ‘conspiracy theories’. The most common themes of misinformation related to skin cancer included assertions of the ‘dangers’ of using sunscreen and alternative sunscreen practices; promotion of tanning and Melanotan (an unlicensed and untested form of α‐melanocyte‐stimulating hormone) as safe practices; claims that risk of skin cancer are limited to people who are older or have fair skin; and assertions of alternative ‘causes’ and alternative ‘cures’ for skin cancer. Sunscreen was particularly vilified as being an ineffective prophylactic measure and a cause of skin cancer. Dermatologists should be aware of misinformation available online relating to skin cancer, and refute and rebut misleading health information.

## Graphical abstract

Misinformation on diseases and treatments is a worldwide threat and can lead to worse outcomes for patients with skin cancer. The aim of this study was to qualitatively assess the content of online misinformation related to skin cancer. The most common themes of misinformation related to skin cancer included the ‘dangers’ of using sunscreen and description of alternative sunscreen practices; tanning as a safe practice; claims that risk of skin cancer is limited to people who are older or have fair skin; alternative ‘causes’ of skin cancer; and alternative ‘cures’ for skin cancer.

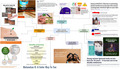

## Introduction

Misinformation has serious ramifications for healthcare and society.^1^ The rise of mobile technology and online self‐publishing has changed how information is communicated and consumed, with emotionally charged narratives from unscientific sources shared rapidly.^1^ Misconceptions about the risk factors, prevention and management of skin cancer continue to interfere with evidence‐based practice.^2^ The aim of this study was to qualitatively assess the content of online misinformation related to skin cancer, identifying primary sources directly from internet search engines and social media, and secondary sources via a literature review on PubMed.

## Report

To identify previously reported misinformation relating to skin cancer available online, a literature review was performed via PubMed using the search terms ‘skin cancer’ OR ‘melanoma’ OR ‘non‐melanoma skin cancer’ OR ‘SCC’ OR ‘BCC’ AND ‘misinformation’ OR ‘disinformation’ OR ‘conspiracy theories’. This search identified 634 abstracts. Following review by two authors (COC, SR), seven papers were considered appropriate for inclusion into the study as they contained content of misinformation related to skin cancer (Table 1). To identify primary sources of misinformation, a Google search was also performed using combinations of these terms. Information was collected from the first 10 pages of results from each Google search. Further targeted searches were performed using YouTube, Facebook, Twitter, TikTok and Instagram, based on the initial Google search. The initial literature review and Google searches were performed in August 2021 and updated in April 2022.

**Table 1:** Summary of the literature review results.

Reference	Content of misinformation
Battie *et al*., 2013^8^	In this US study, 65% of surveyed African Americans never wore sunscreen and > 60% of respondents erroneously believed that they were not at risk for skin cancer
Mosa *et al*., 2019^13^	Families are concerned about malignancy, indicating lingering misinformation or misconception about melanoma risk. Parents listed malignancy as a top reason for wanting the congenital melanocytic naevi removed (37%)
Petukhova *et al*., 2020^10^	A significant number of posts offered medical advice (35%), with the majority of such replies being unsupported by evidence‐based medicine (87%)
Gilhooley *et al*., 2021^6^	Misinformation regarding the safety of Melanotan was commonplace in online discussion forums, by authors without any health credentials
Rafferty *et al*., 2021^9^	Twitter and Facebook posts with negative connotations were exposed to thousands of followers
Vraga *et al*., 2021^12^	Video misinformation heightened beliefs in sunscreen myths and reduced acceptance of sunscreen facts and intentions to wear sunscreen. Real‐time user corrections were partially successful in reducing the effects of the misinformation video on beliefs but not intentions. Exposure to a news literacy video did not inoculate people to misinformation
Tamminga & Lipoff, 2021^3^	Sunscreen‐discouraging posts addressed natural remedies, sunscreen recipes and vitamin D. Comments were twice as likely to discourage photoprotection as to encourage it. Sunscreen‐discouraging posts received more comments

**Figure 1:**
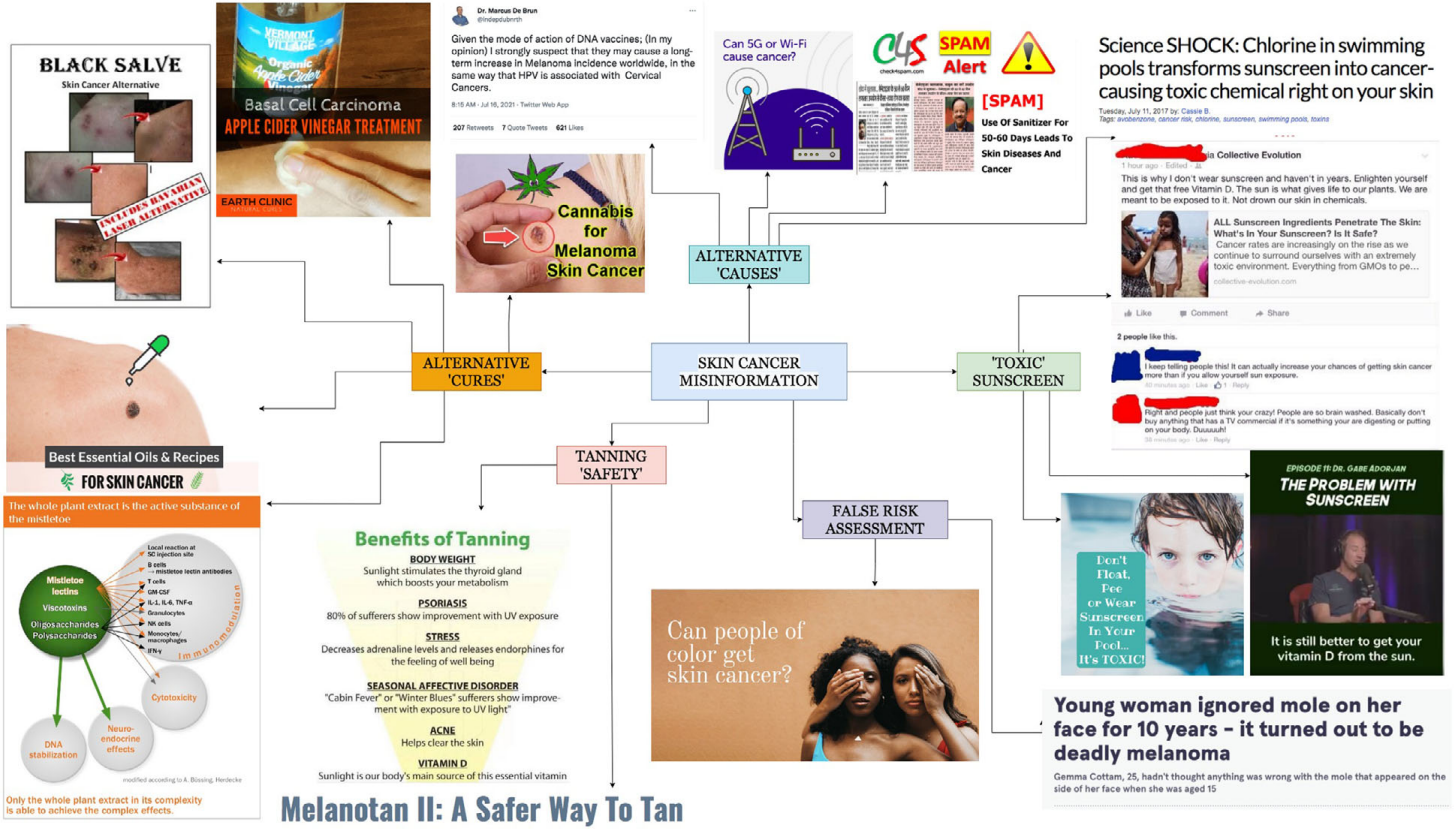
Themes of online misinformation related to skin cancer identified by literature and search engine review. Images, clockwise from top, retrieved from top right: naturalnews.com, facebook.com, youtube.com, chemfreecom.com, mirror.co.uk, solbari.com, melanotan.Eu, facebook.com, integratedmedicine.Co, essentialoilbenefits.com, goodreads.com, earthclinic.com, twitter.com, cannabis.net, twitter.com, twitter.com. [Colour figure can be viewed at wileyonlinelibrary.com]

The most common themes of misinformation related to skin cancer included assertions of the ‘dangers’ of using sunscreen and alternative sunscreen practices; promotion of tanning and Melanotan as safe practices; claims that risk of skin cancer are limited to people who are older or have fair skin; and assertions of alternative ‘causes’ and alternative ‘cures’ for skin cancer (Fig. 1).

Various untrue ‘dangers’ of sunscreen were mentioned, including that sunscreen contains carcinogenic chemicals, and that it causes immune dysfunction and irreversible vitamin D deficiency. Several websites also recommend unsafe sunscreen practices such as natural/homemade sunscreens, avoidance of other photoprotective practices, not requiring sunscreen on cloudy days or on certain body parts, and not requiring reapplication (especially if a sunscreen with a high sun protection factor is used). A study of blog posts about paediatric photoprotection identified that comments were twice as likely to discourage photoprotection as to encourage it, and that posts with negative connotations had higher user engagement.^3^ More seriously, some posts falsely claimed that sunscreen directly causes skin cancer and other cancers, which is ironic given the key role of ultraviolet (UV) radiation in carcinogenesis. Concerns have been raised recently due to reports of toxic effects of oxybenzone and octinoxate on marine ecosystems and the high systemic absorption of sunscreen ingredients. Although the effect on coral is worrying and calls for further research, there is currently no evidence of adverse health outcomes in humans related to sunscreen use.^4^

Tanning was frequently presented as a safe practice that does not increase the risk of skin cancer, with suntan frequently referred to as ‘healthy’. Several websites (often affiliated with indoor tanning companies) suggest that a ‘base tan’ prevents sunburn, and that tanning beds do not pose a risk of skin cancer and are safer than sunbathing. Most YouTube videos on indoor tanning portray it positively and frequently advertise tanning salons.^5^ Melanotan, an unlicensed and untested form of α‐melanocyte‐stimulating hormone, was promoted as a safe way of tanning by avoiding UV radiation.

Given the covert nature of Melanotan use, users often seek access and advice about it on internet forums.^6^ Many influential and apparently authoritative chatroom statements provided peer‐to‐peer assurance regarding the safety of Melanotan and provide advice on dosage, without any supportive scientific evidence, by users without any medical credentials. Although some comments claimed that Melanotan was safer than sunbeds or other forms of UV exposure, Melanotan abuse is strongly associated with use of sunbeds anabolic steroids, especially in the context of bodybuilders preparing for fitness competitions. Melanotan was also promoted as safer than tanning using fake tan, with users claiming that it provided natural protection against sunburn and skin cancer. Many national regulatory agencies for healthcare products have issued safety warnings about the use of Melanotan.^6^ Apart from the intended effect of cutaneous hyperpigmentation, known adverse effects of Melanotan include the development or evolution of pigmented lesions, including multiple reports of melanoma, priapism, infections related to nonsterile water used for reconstitution and blood‐borne infections such as HIV related to needle sharing. The unregulated nature of Melanotan also means that adulteration with other drugs is more likely.

Some websites incorrectly claim that only older adults get skin cancer. However, melanoma is the commonest malignancy overall in people aged 20–30 years, the commonest in men aged < 50 years and the second commonest (after breast cancer) in women < 50 years.^7^ Other websites allege that only people with fair skin get skin cancer and that people who tan easily or have darkly pigmented skin cannot get skin cancer. A recent review of skin cancer in patients with darkly pigmented skin showed that mortality rates in this population are substantially higher than in those with lighter skin tones, as a result of delayed presentation, and that 60% of respondents with darkly pigmented skin erroneously believed that they were not at risk of skin cancer.^8^

Many websites claim that skin cancer is caused by agents other than UV radiation, including sunscreen itself, as discussed previously. Mobile networks, particularly 5G, have been falsely touted as a cause of skin cancer.^9^ During the COVID‐19 pandemic, one website specifically quantified that using hand sanitizer for 50–60 days causes skin cancer. False claims that ‘DNA’ COVID‐19 vaccines cause skin cancer exist on Twitter.

Alternative ‘cures’ for skin cancer are frequently recommended online. One study found that peer‐to‐peer medical advice shared on Facebook groups for individuals who have had Mohs surgery for keratinocyte carcinoma was a source of medical misinformation, including promotion of a substance called ‘Indian black salve’.^10^ Black salve is contains a toxic plant extract (blood root, extracted from *Sanguinaria canadensis*) and is a highly corrosive escharotic, carrying a high risk of infection and permanent disfigurement. Both the US Food and Drug Administration and the American Academy of Dermatology have produced warnings not to use black salve as a treatment for skin cancer. In Germany, mistletoe (*Viscum album*) is commonly taken as an ‘immune booster’ for treatment of melanoma, despite a lack of evidence. Cannabis, either smoked as marijuana or applied topically as cannabis oil, has been promoted as a cure for all forms of skin cancer. Although cannabinoids, the major compounds of the *Cannabis sativa* plant, including the principal compound, tetrahydrocannabinol, have been shown to reduce tumour growth and promote apoptosis and autophagy in melanoma cells *in vivo*,^11^ there are no animal or human studies providing evidence of efficacy in treating skin cancer. Claims that byproducts of acetic acid in apple cider vinegar trigger antitumour properties are published online on some websites. Other alternative ‘cures’ include baking soda (‘a sudden change in pH of the skin tumour stops metastasis’) and essential oils such as black raspberry seed oil, eggplant abstract, frankincense and myrrh oil (‘antioxidants slow the progression of melanoma by improving immunity’). Poultices (also known as cataplasms) are pastes made of herbs, plants and other substances with healing properties, which are recommended to treat skin cancer and ‘get it out by the root’. Particularly concerning is the fact that vitamins, herbal remedies and other ‘all‐natural’ products can also interact with radiotherapy, chemotherapy and immunotherapy for melanoma. High‐dose vitamin A, vitamin C and St John's wort (*Hypericum perforatum*) are particularly problematic.

Misinformation about the risk factors and effective treatments related to skin cancer persists online both in mainstream media and on social media platforms. A recent study showed exposure to news literacy education did not ‘inoculate’ people against misinformation regarding sunscreen myths.^12^ Dermatologists must be aware of the misconceptions regarding skin cancer online and disseminate accurate information to patients. We aim to deeply explore strategies to combat dermatology misinformation in a future article, but the Surgeon General of the USA has provided advice for health professionals and organizations on how to confront health misinformation: (i) proactively engage with patients and the public on health misinformation; (ii) use technology and media platforms to share accurate health information with the public; and (iii) partner with community groups and other local organizations to prevent and address health misinformation.

Brandolini's law states that the amount of effort required to refute misinformation on the internet is far greater than the effort to produce it. Given the potentially lethal consequences of some of the misinformation available online, dermatologists must make the effort to combat misinformation with factual evidence, educate patients on the causes and risk factors for skin cancer, recommend broad‐spectrum sunscreen and other photoprotective practices, and promote evidence‐based treatment for skin cancer.

**Learning points**

• The misinformation culture war has serious ramifications for healthcare and society.
• Misconceptions about the risk factors, prevention and management of skin cancer interfere with evidence‐based practice, with potentially lethal consequences.
• Themes of misinformation related to skin cancer related to skin cancer included assertions of the ‘dangers’ of using sunscreen and alternative sunscreen practices; use of tanning and Melanotan (an unlicensed and untested form of α‐melanocyte‐stimulating hormone) promoted as safe practices; claims that risk of skin cancer are limited to people who are older or have fair skin; and assertions of alternative ‘causes’ and alternative ‘cures’ for skin cancer.
• Sunscreen is particularly targeted by merchants of misinformation online, with extensive reports of lack of efficacy and some reports of direct causation in relation to skin cancer.
• An abundance of ‘natural’ alternative treatments are advocated online, some of which can cause permanent disfigurement or interfere with conventional skin cancer treatment.
• Dermatologists can stop the spread of misinformation relating to skin cancer by educating patients, refuting or rebutting incorrect information, recommending broad‐spectrum sunscreen and other photoprotective practices, and promoting evidence‐based treatments for skin cancer.

## Conflict of interest

The authors declare that they have no conflicts of interest.

## Funding

None.

## Ethics statement

Ethics approval and informed consent not applicable.

## Data availability

Data are available on request from the corresponding author.
